# Psychotherapy for late-life psychopathology – Updates to promote aging in place

**DOI:** 10.3389/fpsyg.2022.994495

**Published:** 2022-09-20

**Authors:** Daniela Aisenberg-Shafran

**Affiliations:** Clinical Psychology of Adulthood and Aging, Ruppin Academic Center, Emek Hefer, Israel

**Keywords:** psychotherapy, aging in place, community, centers, late-life

## Abstract

Psychopathology in late life does not always meet the criterion for a psychiatric diagnosis. Nevertheless, it affects the aging person, their family, employers, and society as a whole. Making psychotherapy accessible for older adults, allowing aging in place, must overcome barriers of mobility, stigma, and emotional difficulty to ask for help. Hence, dedicated counseling and treatment centers should be established in the local authorities for the older adults and their caregivers. Such a local center is described, providing low-cost psychotherapy within an academic environment, accompanied by research to promote suitable therapy of older adults, as well as training programs for professional therapists, not just psychologists, with unique emphasis on late-life psychopathology. This model should be implemented, the more the merrier.

## Introduction

Psychopathology in late life may sometimes look different. The criterion for clinical depression is often not met, but sub-clinical depression occurs more frequently than in the rest of the population (Murri et al., 2022), also related to higher risk of death (Lyness, 2008). Research on late-life depression had shown that psychotherapy is highly efficient in assisting older patients (Tegeler et al., 2020). Psychotherapy was found to have similar effects as pharmacotherapies and with less side effects (Blackburn et al., 2017). Psychological well-being in old age is important both for the aging person and for his/her family that accompanies and suffers psychological distress by itself (Lyness et al., 2009). Furthermore, higher life quality in old age promotes aging in place (Carver et al., 2018) and is important for the welfare and economy of the society. Family members who share the emotional, physical, and even technical process of an aging loved-one, often suffer from psychological distress themselves, in addition to work-day loss for medical visits. This, in addition to health costs for the aging person, also influences the national economy (see Dieleman et al., 2017 for example).

The current healthcare system is not aimed or designed for cost-effectively providing the needs of older individuals living in the community. “Aging in place” is defined as “the ability to live in one’s own home and community safely, independently, and comfortably, regardless of age, income, or ability level” (American Planning Association and the National Association of County and City Health Officials, 2009). That is, aging in place is the accessibility of the necessary resources for older adults to remain in their own environment, maintaining their independence, autonomy, and social network (Wiles et al., 2012).

Most adults age in the community and in fact, there is a very large population that can benefit from psychotherapy at late life, but does not seek treatment, due to a number of barriers:

– Difficulty in mobility, which must be addressed by geographical accessibility of services within the community and not just in hospitals and regional clinics.
– Emotional difficulty in contacting psychiatric departments and clinics associated with mental health.
– Mental difficulty involving misconception and dated perception for psychological treatments and psychotherapy, having negative stereotypes due to cohort and cultural beliefs.

These barriers becoming more and more acknowledgeable (Tegeler et al., 2020). Currently, the common pathways to receive psychotherapy are either by registration in the personal medical file and attending mental health and psychiatry clinics, or by seeking private treatment at a very high cost per session.

## Vision

Accomplishing aging in place requires substantial innovation in the incorporation of advanced technologies, behavioral science, community design, and policy (Kim et al., 2017). Importantly, home health services were indicated as significantly delaying nursing home admission (Chen and Berkowitz, 2012; Young et al., 2015). Hence, dedicated counseling and treatment centers should be established in the local authorities for the older adults and their caregivers. They may not all meet the DSM criterion for a psychiatric diagnosis, but they need a helping hand through the process of their aging.

Such centers should include unique expertise that requires dedicated training. Academic centers where this type of academic training, such as master’s degree programs in clinical psychology of adulthood and aging, should establish a counseling, treatment and research center right on campus. This appealing location may reduce objections and stereotypes and promote referral. The academic setting allows overcoming stigma that creates barriers. That is, when attending a facility within the academy that offers diverse activities (treatment among them), the barrier of entering a mental health hospital or psychiatric clinic is diminished. Such a progress will also contribute to creating a connection between the community and academia, as follows:

1. Providing low-cost professional and accurate psychotherapy for older adults, increasing mental well-being in old age, dealing with life transitions such as retirement, widowhood, decreased health, loss, etc.
2. Providing guidance and support to family members who act as caregivers or accompany the aging process of a parent or spouse.
3. Training professionals and therapists for dedicated work with the old population, based on the extensive knowledge and expertise in the field of treatment for older adults and their family.
4. Conducting up-to-date ecological research on various types of therapy for older adults. Accompanying research should be part of the ongoing process of conducting therapy and management. For example, when providing cognitive behavioral therapy (CBT), relevant frameworks for scientific studies are available and should be implemented. This will contribute to assessing efficiency of treatment and creating more suitable protocols for the older population.

Another possibility is to cooperate with existing treatment services, allowing them to address the older population under the guidance and supervision of relevant professionals in the center, to increase the variety and accessibility of different therapy approaches to the older population.

A counseling and treatment center designed for aging families and an aging population exists in too few nations around the world, and it is important to encourage its establishment as an accessible center in several geographical areas in each country. Such a center is currently being established at the Ruppin Academic Center in Emek Hefer, Israel. The center is located at the country-side Academic Campus, in a non-threatening environment. This center is an accessible and inviting place where older adults, family members and caregivers can come, to enjoy variety of services offered. That is, aside from the psychotherapy provided, the center offers academic courses and participation in academic conferences and activities, as part of being a member and receiving services. Interestingly, groups of older adults, friends, and former colleagues are requesting group attendance to learn various therapeutic techniques, as mindfulness for example.

## Conclusion

Improving the psychological well-being of older adults cannot be put just in the hands of the well-known one-on-one psychotherapy. That is, clinical psychologists should not be the only ones to offer treatment. Working with the multi-generational family requires various professionals to assist in various domains. Social workers, art therapists, nurses, and caregivers should also be part of the treatment team and receive proper training. Moreover, a wider system needs to be involved. This refers to policy makers, national welfare bodies, and region councils, that should invest in making these services accessible, to promote aging in place. This is a world-wide national mission, that requires psycho-education for young, old, caregivers and patients, as well as for authority and policy makers. This is an interdisciplinary effort to make psychotherapy accessible to the pocket and the mind.

## Data availability statement

The original contributions presented in this study are included in the article/supplementary material, further inquiries can be directed to the corresponding author.

## Author contributions

DA-S wrote the text, representing her work as the head department of clinical psychology of adulthood and aging, and leading the foundation of the treatment and counseling center for older adults and their family members in Israel.
